# Prognostic Significance of MicroRNAs in Glioma: A Systematic Review and Meta-Analysis

**DOI:** 10.1155/2019/4015969

**Published:** 2019-03-26

**Authors:** Yanming Zhang, Jigang Chen, Qiang Xue, Junyu Wang, Liang Zhao, Kaiwei Han, Danfeng Zhang, Lijun Hou

**Affiliations:** ^1^Second Sub-Team, Fourth Team, Undergraduate Management Team, Second Military Medical University, Shanghai, China; ^2^Department of Neurosurgery, Changzheng Hospital, Second Military Medical University, Shanghai, China

## Abstract

**Purpose:**

Different microRNAs (miRs) have been demonstrated to relate with the outcome of glioma patients, while the conclusions are inconsistent. We perform a meta-analysis to clarify the relationship between different miRs and prognosis of glioma.

**Methods:**

Related studies were retrieved from PubMed, Embase, and Cochrane Library. Pooled hazard ratios (HRs) of different miRs expression for survival and 95% confidence intervals (CIs) were calculated using random-effects model.

**Results:**

A total of 15 miRs with 4708 glioma patients were ultimately included. Increased expression of miR-15b (HR, 1.584; 95% CI, 1.199-2.092), 21 (HR, 1.591; 95% CI, 1.278-1.981), 148a (HR, 1.122; 95% CI, 1.023-1.231), 196 (HR, 1.877; 95% CI, 1.033-3.411), 210 (HR, 1.251; 95% CI, 1.010-1.550), and 221 (HR, 1.269; 95% CI, 1.054-1.527) or decreased expression of miR-106a (HR, 0.809; 95% CI, 0.655-0.998) and 124 (HR, 0.833; 95% CI, 0.729-0.952) was correlated with poor outcome of glioma patients.

**Conclusions:**

miR-15b, 21, 148a, 196, 210, 221, 106a, and 124 are valuable biomarkers for the prognosis of glioma which might be used in clinical settings.

## 1. Introduction

Central nervous system cancer accounts for 2.3% of all cancer-related mortality worldwide and the annual incidence is reported to be 35 per million individuals [1]. As the most prevalent type of central nervous system cancer, glioma comprises nearly half of malignant brain cancers in adult population [2, 3]. Glioma can be categorized into grades I to IV pathologically according to the World Health Organization (WHO) grading system, and the majority belongs to grade IV, which is known as the most deadly type [4, 5]. In spite of currently available treatment strategies such as surgical resection, adjuvant radiotherapy, and combined radio-chemotherapy, the prognosis of glioma remains pessimistic with its 5-year survival rate being only 2% to 10% [6, 7]. Therefore, identification of prognostic factors is important to clinical decision for proper treatment modality and improvement of long-term outcome.

Advances in studies of genetic biomarkers, such as microRNAs (miRs), have promoted the application of biomarkers in the prognosis of glioma. miRs are a group of short and noncoding RNA molecules and have been identified as the regulators of gene expression [8]. They can work as tumor-suppressing genes as well as oncogenes and thus mediate the progression of cancers [9–11]. Studies show that miRs may be related to the prognosis of different cancers such as lung cancer, gastric cancer, and breast cancer [12–14]. Moreover, the relationships between different kinds of miRs, such as miRNA-15b [15, 16], 21 [17, 18], and 222 [19, 20], and prognosis of glioma have been investigated, while their results are conflicting due to the variability in study design, size of sample, or specimens. Additionally, no systematic review has been performed to explore the role of all pertinent miRs in evaluating glioma prognosis as a whole. In this study, relevant literatures investigating the relationship between numerous kinds of miRs and glioma were systematically reviewed, and pooled results were quantitatively analyzed to evaluate the prognostic value of different miRs in glioma.

## 2. Materials and Methods

### 2.1. Search Strategy

The meta-analysis was conducted in line with the recommendations of Meta-Analysis of Observational Studies in Epidemiology group (MOOSE) [21] and Preferred Reporting Items for Systematic Reviews and Meta-Analysis: The PRISMA Statement [22]. Three databases including PubMed, Embase, and Cochrane Library were searched for studies examining the relationships between miRs and prognosis of glioma by two authors (Danfeng Zhang and Qiang Xue) independently on August 8^th^, 2017 without date limit. We restricted the language to English. The Mesh terms were defined as “microrna/micrornas/mirna/miRs” with “gliomas/glial cell tumor/glioblastoma” in the search process. The reference lists of retrieved articles were also checked for pertinent literatures. The complete search strategy for PubMed and Cochrane Library was presented in Supplementary Material.

### 2.2. Inclusion Criteria

Studies were included in this meta-analysis if they (1) recruited patients of glioma; (2) measured the expression of miRs in tumor tissue, serum, or plasma, as well as the survival prognosis of patients; (3) reported the survival curves for overall survival (OS) or disease-free survival (DFS) or cause-specific survival (CSS) or recurrence-free survival (RFS) with or without the hazard ratio (HR) and its 95% confidence intervals (CIs).

### 2.3. Exclusion Criteria

We excluded studies if (1) they were letters, reviews, or experimental studies; (2) the number of articles examining the relationship between miRs and glioma was less than three; (3) the original data could not be pooled. If one cohort was reported in two or more articles, we included the study most fully adjusted in order to prevent the disturbance of confounders.

### 2.4. Data Extraction and Quality Assessment

Study characteristics and original data were collected by three authors (Yanming Zhang, Qiang Xue, and Jigang Chen), including first author's name, publication year, study design, study population, size of population, age and sex of participants, follow-up duration, type of sample, method of measuring miRs expression, and HRs and their 95% CIs. If HRs and 95% CIs were not reported in the included articles, we estimated them from Kaplan-Meier survival curves with methods described by Tierney et al. using Engauge Digitizer version 4.1 [23]. If only HRs and P values were reported, we estimated the 95% CIs using previously described method [24].

Studies were included according to the following checklist on the basis of the criteria provided by MOOSE group [21]: clearly defined study design; clearly described study population (country); sufficiently large sample (N>30); clearly described outcome (OS, CSS, DFS, or RFS); clear defined miRs measurement, including quantitative real-time polymerase chain reaction (qRT-PCR) or in situ hybridization (ISH); clear definition of cut-off values; miRs measurement in tumor tissue, plasma, or serum; sufficiently long follow-up. Studies were excluded if they did not meet these criteria. Quality of included studies was systematically evaluated according to Newcastle-Ottawa Scale by two reviewers (Liang Zhao and Danfeng Zhang) independently [25]. Disagreement was solved by joint review.

### 2.5. Statistical Analysis

HRs and their 95% CIs extracted from studies were pooled using Stata version 12.0 (StataCorp, College Station, Texas, USA) and random-effects model. We used Chi-square test and* I*^*2*^ statistic in the assessment of heterogeneities among studies, and* I*^*2*^ values of <40%, 40%-75%, and >75% were defined as low, moderate, and high, respectively [26]. Subgroups analysis was conducted according to the type of survival prognosis (OS versus DFS) and data sources (direct extraction versus calculation from HR and P versus calculation from survival curve). In the pooled analysis, Egger's test was employed in the analysis of publication bias. Sensitivity analysis was conducted by the removal of individual study by turns. P<0.1 was considered as significant in the analysis of publication bias and heterogeneity, while a significant level of 0.05 was used in other analyses.

## 3. Results

### 3.1. Study Selection

The study selection process was shown in Figure I of Supplementary Material. A total of 2470 records were available in the initial search, including 1160 records from PubMed, 1294 from Embase, and 16 from Cochrane Library. 1837 studies remained for full texts review after removing the duplicates and reviewing the abstract. No eligible study was detected by screening the reference lists. Finally, 31 studies met the inclusion criteria and were included in our meta-analysis.

### 3.2. Study Characteristics

The quality assessment of each study was shown in Table I of Supplementary Material. The number of literatures evaluating the association between miRs and the prognosis of glioma were listed in Table II of Supplementary Material. The main characteristics of included articles were described in Table 1. All of them were retrospective and published between 2010 and 2017. A total of 4708 glioma patients were evaluated for the prognostic value of 15 different miRs, with a median sample size of 109 patients (range, 38–548 patients). Expression of miRs was mainly measured in tumor tissues, while four studies examined miRs in serum or plasma [36, 41, 47, 49]. Most studies used qRT-PCR to detect miRs, while three employed ISH and microarray [18, 31, 41]. HRs and 95% CIs were not reported in 14 studies [15, 16, 20, 27, 30, 32, 33, 37, 39, 42, 43, 45, 46, 48], and we estimated them by methods described above. The cutoff value was not reported in 11 articles [15, 18, 33, 36, 38, 40, 45–48, 50]. The reported HRs were adjusted for related variables such as pathological grade, Karnofsky performance score (KPS) and tumor size in nine studies [17–19, 28, 29, 34, 38, 41, 49] (Table 1).

### 3.3. Meta-Analysis

The pooled HRs together with the heterogeneity for all miRs were demonstrated in Table 2.

### 3.4. High Expression of miR-15b, 21, 148a, 196, 210, and 221 Predicts Poor Prognosis in Glioma Patients

**Figure 1:**
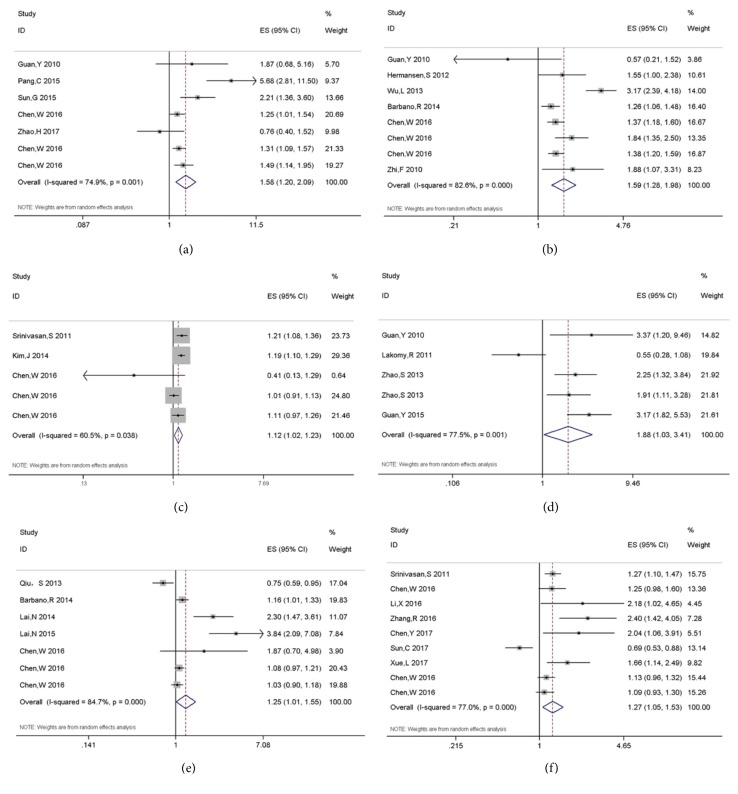
Forest plots of miR-15b (a), 21 (b), 148a (c), 196 (d), 210 (e), and 221 (f) and glioma prognosis.

Five studies were included to investigate the relationship between high expression of miR-15b and DFS/OS [15, 16, 34, 37, 41]. The pooled results indicated that high miR-15b expression was significantly associated with the poor prognosis in glioma (HR, 1.584; 95% CI, 1.199-2.092, P=0.001, Figure 1(a)).

Six studies examined the prognostic value of miR-21 in glioma [17, 18, 29, 34, 37, 38], suggesting that miR-21 overexpression significantly predicted poor prognosis in glioma (HR, 1.591; 95% CI, 1.278-1.981, P<0.001, Figure 1(b)).

Three literatures focused on the relationship between high expression of miR-148a and OS/DFS [30, 34, 50]. The summary results suggested that miR-148a was correlated with shorter DFS/OS (HR, 1.122; 95% CI, 1.023-1.231, P=0.015, Figure 1(c)).

Pooled results also demonstrated significant relationship between miR-196, 210, and 221 and poor prognosis in glioma (HR, 1.877; 95% CI, 1.033-3.411, P=0.039 for miR-196; HR, 1.251; 95% CI, 1.010-1.550, P=0.04 for miR-210; HR, 1.269; 95% CI, 1.054-1.527, P=0.012 for miR-221; Figures 1(d)–1(f)).

### 3.5. Low Expression of miR-106a and 124 Predicts Poor Prognosis in Glioma Patients

**Figure 2:**
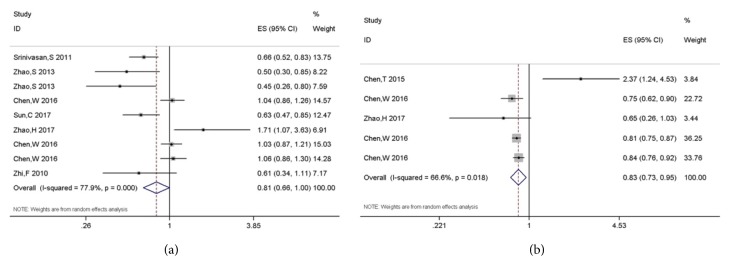
Forest plots of miR-106a (a) and 124 (b) and glioma prognosis.

There were six [20, 29–31, 34, 41] and three [34, 41, 45] studies investigating the prognostic value of miR-106a and miR-124 in glioma, respectively. The summary HRs indicated these two miRs were negatively associated with poor prognosis in glioma (HR, 0.809; 95% CI, 0.655-0.998, P=0.048 for miR-106a; HR, 0.833; 95% CI, 0.729-0.952, P=0.007 for miR-124, Figures 2(a)-2(b)).

### 3.6. No Significant Relationship between Overexpression of miR-10b, 17, 20a, 155, 182, 200b, and 222 and Poor Prognosis in Glioma Patients

**Figure 3:**
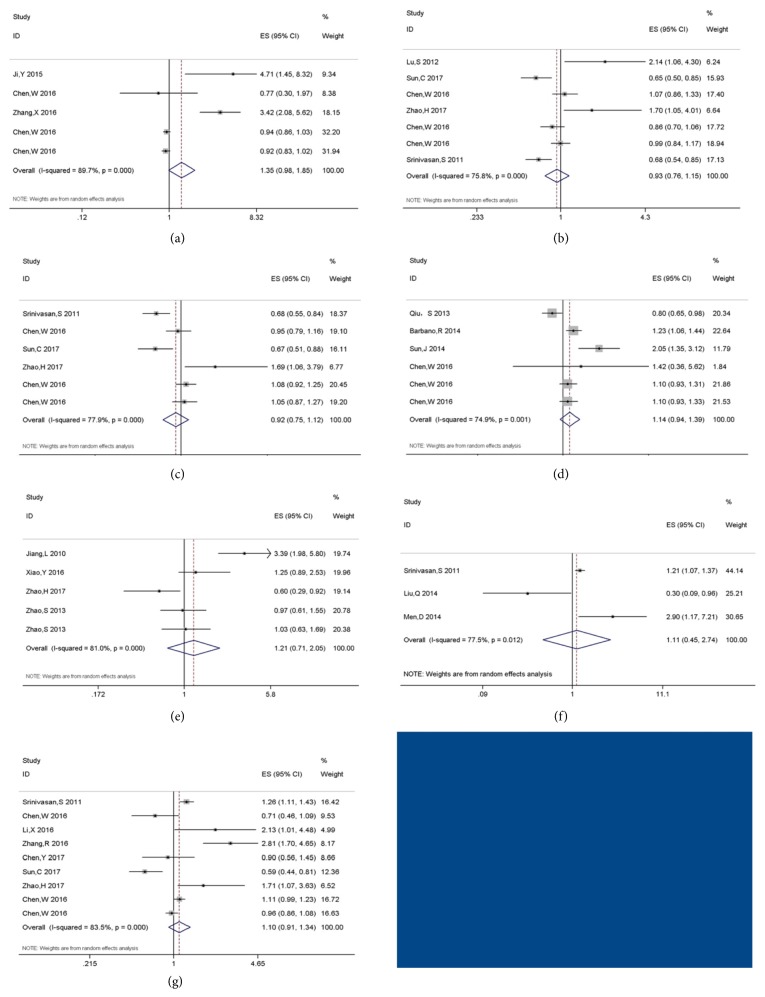
Forest plots of miR-10b (a), 17 (b), 20a (c), 155 (d), 182 (e), 200b (f), and 222 (g) and glioma prognosis.

Several different studies were included to examine the prognostic value of miR-10b, 17, 20a, 155, 182, 200b, and 222 in glioma. However, pooled HRs suggested no statistical relationships between these miRs and prognosis of glioma. The detailed results were illustrated in Table 2 and Figure 3.

### 3.7. Subgroups Analysis

In the subgroup of OS outcomes, we found high expression of miR-10b predicted poor prognosis in glioma patients (HR, 3.70; 95% CI, 2.40-5.70, P<0.05) (Table III in Supplementary Material). For data calculation from HR and P value, we detected that low expression of miR-17 and 20a was associated with poor prognosis in glioma patients (HR, 0.67, 95% CI, 0.56-0.79, P<0.05 for miR-17; HR, 0.68, 95% CI, 0.57-0.80, P<0.05 for miR-20a, Table III in Supplementary Material). For data calculation from survival curve, overexpression of miR-10b and 182 was detected to be related to poor prognosis after glioma (HR, 3.42, 95% CI, 2.08-5.62, P<0.05 for miR-10b; HR, 3.39, 95% CI, 1.98-5.80, P<0.05 for miR-182, Table III in Supplementary Material).

### 3.8. Publication Bias

Publication bias was assessed for the meta-analysis of all miRs and we found no publication bias by Egger's test, which was shown in Table 2.

### 3.9. Sensitivity Analysis

We have done the sensitivity analysis through removing studies one by one in the analysis of all miRs. Our results were roughly not altered suggesting that our pooled HRs and the 95%CIs were basically stable. However, when it went to miR-10b, the result turned to be significant, suggesting that high miR-10b expression was associated with the poor prognosis in glioma if we removed data from Chen et al.'s article (HR, 1.428; 95% CI, 1.022-1.995, P=0.037) [34]. For miR-155, high miR-155 expression was associated with the poor prognosis in glioma (HR,1.22; 95% CI, 1.044-1.425, P=0.012) after removing Qiu et al.'s study [28].

## 4. Discussion

Mounting evidences have shown that various miRs are related to the survival outcome in glioma patients. However, different studies present with inconsistent conclusions. For example, three studies investigate the association between miR-10b and glioma prognosis, and the results are significant in Zhang et al. and Ji et al. [46, 82] and insignificant in Chen et al. [34]. Similar conflicting results are also demonstrated in researches exploring other miRs [13, 36, 83–85]. Therefore, it is crucial to perform current meta-analysis to have an overall understanding of relationships between miRs expression and prognosis of glioma patients.

A total of 15 miRs and their ability in predicting prognosis of glioma are investigated in this study. Patients with high levels of miR-15b, 21, 148a, 196, 210, and 221 expression have a statistically significant poorer DFS/OS than those with low expression levels. Contrastively, decreased expression of miR-106a and 124 is associated with poor prognosis in patients of glioma. There are some other miRs including miR-10b, 17, 20a, 155, 182, 200b, and 222 which are indicated to have no prognostic value in glioma. There is no publication bias after the assessment using Egger's test and the pooled HRs remain the same when removing studies one by one.

Among the miRs whose overexpression is indicated to be associated with poor DFS/OS, miR-21 is the first discovered microRNA and known to be widely expressed in human tissues. It is also the most studied tumor-related biomarkers and might play an essential role in many different cancers [86]. Increased expression level of miR-21 has been discovered to be related to dismal outcome in cancer patients [13]. miR-21 is indicated to be overly expressed in glioma in a WHO-grade specific manner [54]. Several literatures assure that miR-21 can induce the tumor growth, invasion, and migration and inhibit cell apoptosis [55, 87]. miR-21 has been identified to target at the tumor suppressing genes, such as the protein tyrosine phosphatase (PTEN), programmed cell death 4 (PDCD4), and B cell translocation gene 2 (BTG2). Inhibition of miR-21 would lead to the upregulation of these genes, which ultimately affect the cancer progression and prognosis [13, 66, 88–90].

Only two miRs (miR-106a and miR-124) are proved in this study that their downregulations are connected with poor prognosis. As a protective microRNA, miR-106a is located at Xq26.2 and the length of mature miR-106a is 23 nucleotides. Previous study has shown that miR-106a has a cancer suppressing effect through antiproliferation and inducing apoptosis in glioma cells. This effect might arise from E2F1 inhibition via posttranscriptional regulation [91]. Similarly, miR-124 is reported to have effects of tumor suppression via the regulation of cell proliferation, apoptosis, migration, and invasion in certain cancer diseases [67, 92, 93]. Study also indicated that miR-124 works through the inhibition of STAT3 signal to enhance the T cell mediated clearance of glioma cells [70].

The relationships between overexpression of seven miRs (miR-10b, 17, 20a, 155, 182, 200b, and 222) and DFS/OS for glioma patients were not proved in our study. This might attribute to the nature of miRs themselves. For example, miR-17 is extensively studied, and it proves to have both the tumor suppressing and oncogenic functions. Upregulation of miR-17 can promote cancer growth via aiming E2F1 and increase angiogenesis through thrombospondin-1 [71]. Contrastively, overexpression of miR-17 can also lead to the decreased cell migration and proliferation by the repressing of fibronectin expression [94]. Moreover, different sample size, type of specimens, and prognosis assessment might also produce the inconsistent conclusions which ultimately lead to the insignificant results in our meta-analysis. Summary of miRs along with altered expression and potential targets as well as pathways in this study is listed in Table 3.

Though the measurement of miRs expression levels is a convenient way in predicting the glioma prognosis, difficulties still exist before applying miRs in the clinical settings. First, cell-free miRs would release from some normal human tissues as well and might interfere the final results to some degree [95]. Therefore, it is important to determine the source of tumor-specific miRs and create a method which could differentiate cancer population from healthy group. Second, no standard procedure for the measurement of miRs has been confirmed, which might be the source of contradictory results. Moreover, a single microRNA can be associated with different tumor tissues. For example, the prognostic value of miR-21 has been established among the patients of breast cancer [96], pancreatic ductal adenocarcinoma [97], and gastric cancer [98]. Therefore, a group of miRs specific to glioma is useful and might significantly improve the prognostic accuracy [35].

To our knowledge, though some meta-analyses regarding the prognostic value of several miRs in glioma patients have been published [36, 83–85], these studies are incomplete and avoid assessing some other available miRs. We include all of the miRs which have been explored previously, and a total of 15 miRs are investigated ultimately. Among these 15 miRs, eight of them have established the prognostic significance with glioma. However, relationship between the remaining miRs and prognosis of glioma patients should be validated by further large-scale prospective studies in future. Our study also has advantages in including the newly published trials from different places and times, which are representative enough.

Limitations of our meta-analysis should be noticed before interpreting the results. Firstly, as we mentioned above, there is no single microRNA which is specific to glioma exclusively, and the panel of miRs which can be used to distinguish glioma from other cancers and satisfactorily predict the prognosis has not been discovered yet. Therefore, the clinical application of miRs is restricted. Secondly, the heterogeneity among studies is generally significant. Thirdly, the prognosis is evaluated by different indicators, such as the overall survival and disease-free survival, which might be the source of heterogeneity. Fourthly, all the included literatures are retrospective and there lack relative high-quality trials. Lastly, the number of available studies is limited for some miRs and it might be insufficient to draw a definite conclusion.

## Figures and Tables

**Table 1:** Characteristics of articles with Kaplan-Meier survival curves in glioma.

microRNA	Study	Country	Study design	Sample	Number	Stage	Cut-off	Follow-up (months)	Result	HR(H/L)	95%CI	p
10b	Ji, Y 2015	China	R	Frozen	95	I-IV	Median	60	OSm	4.71	1.45-8.32	<0.001
10b	Chen, W 2016	TCGA	R	Tissue	109	I-IV	Median	>60	DFSu	1.09	0.98-1.21	0.12
10b	Zhang, X 2016	China	R	Frozen	128	I-IV	None	80	OSu	3.42	2.08-5.62	<0.001
10b	Chen, W 2016	TCGA	R	Tissue	109	I-IV	Median	>60	DFSu	1.30	0.53-3.2	0.58
10b	Chen, W 2016	TCGA	R	Tissue	109	I-IV	Median	>60	DFSu	1.06	0.97-1.15	0.18
15b	Guan, Y 2010	Japan	R	Frozen	39	I-IV	Mean	>60	OSm	1.87	0.68-5.16	0.227
15b	Pang, C 2015	China	R	Frozen	76	II-IV	None	>60	OSu	5.68	2.81-11.50	<0.001
15b	Sun, G 2015	China	R	Frozen	92	I-IV	Median	>60	OSu	2.21	1.36-3.6	0.001
15b	Chen, W 2016	TCGA	R	Tissue	109	I-IV	Median	>60	DFSu	0.67	0.51-0.87	0.003
15b	Zhao, H 2017	America	R	Serum	106	I-IV	Median	24	OSu	0.76	0.40-1.52	0.028
15b	Chen, W 2016	TCGA	R	Tissue	109	I-IV	Median	>60	DFSu	0.80	0.65-0.99	0.04
15b	Chen, W 2016	TCGA	R	Tissue	109	I-IV	Median	>60	DFSu	0.76	0.63-0.91	0.003
17	Lu, S 2012	China	R	Tissue	108	I-IV	Median	>100	OSm	2.14	1.06-4.30	0.034
17	Sun, C 2017	TCGA	R	Tissue	548	I-IV	Median	130	OSu	0.6517	0.50-0.85	0.002
17-5b	Chen, W 2016	TCGA	R	Tissue	109	I-IV	Median	>60	DFSu	0.934	0.76-1.16	0.54
17-5b	Zhao, H 2017	America	R	Serum	106	I-IV	Median	24	OSu	1.7	1.05-4.01	0.043
17-5b	Chen, W 2016	TCGA	R	Tissue	109	I-IV	Median	>60	DFSu	1.16	0.95-1.42	0.15
17-5b	Chen, W 2016	TCGA	R	Tissue	109	I-IV	Median	>60	DFSu	1.01	0.80-1.28	0.94
17-5p	Srinivasan, S 2011	TCGA	R	Tissue	111	I-IV	60th percentile	120	OSm	0.68	0.54-0.85	0.0008
20a	Srinivasan, S 2011	TCGA	R	Tissue	111	I-IV	60th percentile	120	OSm	0.68	0.55-0.84	<0.001
20a	Chen, W 2016	TCGA	R	Tissue	109	I-IV	Median	>60	DFSu	0.95	0.79-1.14	0.59
20a	Sun, C 2017	TCGA	R	Tissue	548	I-IV	Median	130	OSu	0.6708	0.51-0.88	0.005
20a	Zhao, H 2017	America	R	Serum	106	I-IV	Median	24	OSu	1.69	1.06-3.79	0.04
20a	Chen, W 2016	TCGA	R	Tissue	109	I-IV	Median	>60	DFSu	1.05	0.87-1.27	0.63
20a	Chen, W 2016	TCGA	R	Tissue	109	I-IV	Median	>60	DFSu	0.93	0.80-1.08	0.35
21	Guan, Y 2010	Japan	R	Frozen	39	I-IV	Mean	>60	OSm	0.57	0.21-1.52	0.264
21	Hermansen, S 2012	Denmark	R	FFPE	189	I-IV	None	>60	OSm	1.545	1.002-2.381	0.049
21	Wu, L 2013	China	R	Frozen	152	I-IV	Mean	60	OSm	3.17	2.39-4.179	<0.001
21	Barbano, R 2014	TCGA	R	Tissue	191	I-IV	None	>110	OSu	1.26	1.06-1.48	0.007
21	Chen, W 2016	TCGA	R	Tissue	109	I-IV	Median	>60	DFSu	0.73	0.58-0.91	0.006
21	Chen, W 2016	TCGA	R	Tissue	109	I-IV	Median	>60	DFSu	0.54	0.37-0.80	0.002
21	Chen, W 2016	TCGA	R	Tissue	109	I-IV	Median	>60	DFSu	0.72	0.56-0.92	0.009
21	Zhi, F 2010	China	R	Tissue	124	I-IV	Median	100	OSm	1.882	1.07-3.308	0.028
106a	Srinivasan, S 2011	TCGA	R	Tissue	111	I-IV	60th percentile	120	OSm	0.66	0.52-0.83	<0.001
106a	Zhao, S 2013	China	R	FFPE	114	I-IV	Median	50	OSm	0.504	0.297–0.854	0 .011
106a	Zhao, S 2013	China	R	FFPE	103	I-IV	Median	50	OSm	0.452	0.255–0.800	0 .006
106a	Chen, W 2016	TCGA	R	Tissue	109	I-IV	Median	>60	DFSu	0.94	0.73-1.20	0.62
106a	Sun, C 2017	TCGA	R	Tissue	548	I-IV	Median	130	OSu	0.6341	0.47-0.85	0.003
106a	Zhao, H 2017	America	R	Serum	106	I-IV	Median	24	OSu	1.71	1.07-3.63	0.038
106a	Chen, W 2016	TCGA	R	Tissue	109	I-IV	Median	>60	DFSu	0.96	0.80-1.15	0.67
106a	Chen, W 2016	TCGA	R	Tissue	109	I-IV	Median	>60	DFSu	0.97	0.81-1.17	0.76
106a	Zhi, F 2010	China	R	Tissue	124	I-IV	Median	100	OSm	0.6139	0.34-1.11	0.108
124	Chen, T 2015	China	R	Frozen	137	I-IV	None	60	OSm	2.37	1.24-4.528	0.009
124	Chen, W 2016	TCGA	R	Tissue	109	I-IV	Median	>60	DFSu	1.19	1.06-1.33	0.003
124	Zhao, H 2017	America	R	Serum	106	I-IV	Median	24	OSu	0.65	0.26-1.03	0.062
124	Chen, W 2016	TCGA	R	Tissue	109	I-IV	Median	>60	DFSu	1.33	1.08-1.64	0.007
124	Chen, W 2016	TCGA	R	Tissue	109	I-IV	Median	>60	DFSu	1.23	1.11-1.36	<0.001
148a	Srinivasan, S 2011	TCGA	R	Tissue	111	I-IV	60th percentile	120	OSm	1.21	1.08-1.356	0.001
148a	Kim, J 2014	TCGA	R	Tissue	482	I-IV	None	>60	OSu	1.19	1.10-1.29	<0.001
148a	Chen, W 2016	TCGA	R	Tissue	109	I-IV	Median	>60	DFSu	0.9	0.79-1.03	0.13
148a	Chen, W 2016	TCGA	R	Tissue	109	I-IV	Median	>60	DFSu	2.44	0.77-7.74	0.13
148a	Chen, W 2016	TCGA	R	Tissue	109	I-IV	Median	>60	DFSu	0.99	0.90-1.09	0.85
155	Qiu, S 2013	TCGA	R	Tissue	480	I-IV	50th percentile	>100	OSm	0.796	0.646-0.982	0.033
155	Barbano, R 2014	TCGA	R	Tissue	191	I-IV	None	>110	OSu	1.23	1.06-1.44	0.008
155	Sun, J 2014	China	R	Tissue	131	I-IV	Mean	80	OSu	2.05	1.35-3.12	<0.001
155	Chen, W 2016	TCGA	R	Tissue	109	I-IV	Median	>60	DFSu	0.91	0.77-1.07	0.27
155	Chen, W 2016	TCGA	R	Tissue	109	I-IV	Median	>60	DFSu	0.70	0.18-2.73	0.62
155	Chen, W 2016	TCGA	R	Tissue	109	I-IV	Median	>60	DFSu	0.91	0.77-1.07	0.26
182	Jiang, L 2010	China	R	FFPE	119	I-IV	Median	80	OSm	3.39	1.98-5.80	<0.001
182	Xiao, Y 2016	China	R	Blood	112	I-IV	None	60	OSm	1.25	0.89-2.53	0.013
182	Zhao, H 2017	America	R	Serum	106	I-IV	Median	24	OSu	0.6	0.29-0.92	0.037
182a	Zhao, S 2013	China	R	FFPE	114	I-IV	Median	50	OSm	0.974	0.611–1.554	0 .912
182a	Zhao, S 2013	China	R	FFPE	103	I-IV	Median	50	OSm	1.032	0.630–1.693	0 .900
196	Guan, Y 2010	Japan	R	Frozen	39	I-IV	Mean	>60	OSm	3.37	1.20-9.46	0.021
196	Lakomy, R 2011	Czech	R	FFPE	38	I-IV	Median	>60	OSu	0.547	0.2776-1.0776	0.049
196a	Zhao, S 2013	China	R	FFPE	114	I-IV	Median	50	OSm	2.252	1.321-3.841	0.003
196a	Zhao, S 2013	China	R	FFPE	103	I-IV	Median	50	OSm	1.906	1.108-3.281	0.021
196a	Guan, Y 2015	China	R	Frozen	63	I-IV	None	>60	OSu	3.17	1.82-5.53	0.007
200b	Srinivasan, S 2011	TCGA	R	Tissue	111	I-IV	60th percentile	120	OSm	1.21	1.067-1.372	0.003
200b	Liu, Q 2014	China	R	Tissue	73	I-IV	None	40	OSu	0.3	0.09-0.96	0.05
200b	Men, D 2014	China	R	Frozen	266	I-IV	Median	60	OSm	2.9	1.166-7.21	0.022
210	Qiu, S 2013	TCGA	R	Tissue	480	I-IV	50th percentile	>100	OSm	0.749	0.591-0.949	0.017
210	Barbano, R 2014	TCGA	R	Tissue	191	I-IV	None	>110	OSu	1.16	1.01-1.33	0.038
210	Lai, N 2014	China	R	Frozen	125	I-IV	Mean	>100	OSu	2.3	1.47-3.61	0.0003
210	Lai, N 2015	China	R	Serum	126	I-IV	Mean	>80	OSm	3.84	2.09-7.08	<0.001
210	Chen, W 2016	TCGA	R	Tissue	109	I-IV	Median	>60	DFSu	0.97	0.83-1.14	0.71
210	Chen, W 2016	TCGA	R	Tissue	109	I-IV	Median	>60	DFSu	0.53	0.20-1.43	0.21
210	Chen, W 2016	TCGA	R	Tissue	109	I-IV	Median	>60	DFSu	0.93	0.84-1.03	0.17
221	Srinivasan, S 2011	TCGA	R	Tissue	111	I-IV	60th percentile	120	OSm	1.27	1.097-1.471	0.001
221	Chen, W 2016	TCGA	R	Tissue	109	I-IV	Median	>60	DFSu	0.92	0.79-1.06	0.26
221	Li, X 2016	China	R	Tissue	45	I-IV	Mean	36	OSu	2.18	1.02-4.65	0.044
221	Zhang, R 2016	China	R	Blood	50	I-IV	None	50	OSu	2.4	1.42-4.05	0.001
221	Chen, Y 2017	China	R	Tissue	114	I-IV	None	72	OSm	2.039	1.06-3.91	0.032
221	Sun, C 2017	TCGA	R	Tissue	548	I-IV	Median	130	OSu	0.6856	0.53-0.88	0.003
221	Xue, L 2017	China	R	Tissue	165	I-IV	Median	60	OSu	1.656	1.135-2.486	0.009
221	Chen, W 2016	TCGA	R	Tissue	109	I-IV	Median	>60	DFSu	0.80	0.19-3.30	0.77
221	Chen, W 2016	TCGA	R	Tissue	109	I-IV	Median	>60	DFSu	0.88	0.74-1.04	0.14
222	Srinivasan, S 2011	TCGA	R	Tissue	111	I-IV	60th percentile	120	OSm	1.26	1.11-1.43	0.0004
222	Chen, W 2016	TCGA	R	Tissue	109	I-IV	Median	>60	DFSu	1.04	0.92-1.18	0.53
222	Li, X 2016	China	R	Tissue	45	I-IV	Mean	36	OSu	2.13	1.01-4.48	0.043
222	Zhang, R 2016	China	R	Blood	50	I-IV	None	50	OSu	2.81	1.70-4.65	0.0004
222	Chen, Y 2017	China	R	Tissue	114	I-IV	None	72	OSm	0.899	0.559-1.447	0.661
222	Sun, C 2017	TCGA	R	Tissue	548	I-IV	Median	130	OSu	0.5947	0.44-0.81	0.001
222	Zhao, H 2017	America	R	Serum	106	I-IV	Median	24	OSu	1.71	1.07-3.63	0.038
222	Chen, W 2016	TCGA	R	Tissue	109	I-IV	Median	>60	DFSu	1.41	0.91-2.17	0.12
222	Chen, W 2016	TCGA	R	Tissue	109	I-IV	Median	>60	DFSu	0.90	0.80-1.01	0.07

CI: confidence interval; DFS: disease free survival; FFPE: formalin-fixed paraffin-embedded; HR (H/L): hazard ratio (High/Low); OS: overall survival; R: retrospective; TGGA: The Cancer Genome Atlas; m: multivariate analysis; u: univariate analysis.

**Table 2:** Summary of the HR for microRNA expression in glioma.

microRNA	Survival analysis	Number of articles	Included references	HR	95%CI	P value	*∗*Heterogeneity	Total patients	Figure	Publication bias
10b	OS/DFS	3	[27–29]	1.349	0.984-1.849	0.063	89.7%, p<0.001	550	4A	0.16
15b	OS/DFS	5	[15, 16, 26, 28, 30]	1.584	1.199-2.092	0.001	74.9%, p=0.001	640	2A	0.263
17	OS/DFS	5	[20, 26, 28, 31, 32]	0.933	0.759-1.149	0.516	75.8%, p<0.001	1200	4B	0.368
20a	OS/DFS	4	[20, 26, 28, 31]	0.919	0.755-1.119	0.399	77.9%, p<0.001	1092	4C	0.925
21	OS/DFS	6	[18, 28, 30, 33–35]	1.591	1.278-1.981	<0.001	82.6%, p<0.001	1022	2B	0.536
106a	OS/DFS	6	[20, 26, 28, 31, 34, 36]	0.809	0.655-0.998	0.048	77.9%, p<0.001	1433	3A	0.177
124	OS/DFS	3	[26, 28, 37]	0.833	0.729-0.952	0.007	66.6%, p=0.018	570	3B	0.516
148a	OS/DFS	3	[28, 31, 38]	1.122	1.023-1.231	0.015	60.5%, p=0.038	920	2C	0.254
155	OS/DFS	4	[28, 33, 39, 40]	1.143	0.942-1.387	0.175	74.9%, p=0.001	1129	4D	0.586
182	OS	4	[26, 36, 41, 42]	1.206	0.709-2.051	0.489	81%, p<0.001	554	4E	0.955
196	OS	4	[30, 36, 43, 44]	1.877	1.033-3.411	0.039	77.5%, p=0.001	357	2D	0.893
200b	OS	3	[31, 45, 46]	1.113	0.451-2.744	0.816	77.5%, p=0.012	450	4F	0.923
210	OS/DFS	5	[28, 33, 40, 47, 48]	1.251	1.010-1.550	0.04	84.7%, p<0.001	1249	2E	0.181
221	OS/DFS	7	[19, 20, 28, 31, 49–51]	1.269	1.054-1.527	0.012	77.0%, p<0.001	1360	2F	0.194
222	OS/DFS	7	[19, 20, 26, 28, 31, 49, 50]	1.104	0.907-1.343	0.325	83.5%, p<0.001	1301	4G	0.765

DFS: disease free survival; HR: hazard ratio; OS: overall survival; *∗*Higgins *I*^*2*^ statistic.

**Table 3:** Summary of miRs with altered expression, their potential targets, and pathways entered this study.

miRNA	Expression	Potential targets	Pathways	Reference
15b	Up	cyclin D1, MMP-3, NRP	Angiogenesis, cell apoptosis, cell cycle progression, cell invasion	[52, 53]
21	Up	BTG2, PDCD4, PTEN	Cell apoptosis, invasion, migration, tumor growth	[54–56]
148a	Up	BIM, MIG6	Cell apoptosis	[38]
196	Up	HOXA7, HOXB8, HOXC8, HOXD8, I*κ*B*α*	Malignant transformation, tumorigenesis,	[57–60]
210	Up	FGFRL1, HIF-1a	Angiogenesis, cell migration, cell proliferation,	[61–63]
221	Up	AKT, p27Kipl, Growth factor signaling pathways	Cell proliferation, cell apoptosis, malignant phenotype	[64, 65]
106a	Down	E2F1, TIMP-2	Cell apoptosis, cell invasion, cell proliferation,	[66]
124	Down	STAT3	T cell mediated clearance of glioma	[67]
10b	Up	RhoC, uPA	Cell invasion, cell migration	[68, 69]
17	Up or down	E2F1, TSP-1	Angiogenesis, cell growth, cell migration	[70, 71]
20a	Up	E2F1, TIMP-2	Cell invasion, cell proliferation	[72–75]
155	Up	FOXO3a, p53	Cell invasion, cell migration	[76–78]
182	Down	FOXO3, MITF-M	Cell migration, cell survival	[79]
200b	Up or down	cyclin D1, EGFR, RND3	Cell migration, epithelial-to mesenchymal transition	[80, 81]
222	Up	p27Kip1	Cell cycle progression, cell invasion, cell proliferation	[19, 65]

AKT, AKT serine/threonine kinase; BTG2, B cell translocation gene 2; E2F1, E2F transcription factor 1; EGFR, epidermal growth factor receptor; FGFRL1, fibroblast growth factor receptor-like 1; FOXO3a, forkhead box O3; HIF-1a, hypoxia-inducible factor 1a; HOX, homeobox; MIG6, mitogen-inducible gene 6; MITF-M, microphthalmia-associated transcription factor-M; MMP-3, matrix metalloproteinase-3; NRP, nitrogen regulatory protein; PDCD4, programmed cell death 4; PTEN, protein tyrosine phosphatase; RHOC, ras homolog family member C; RND3, rho family GTPase 3; STA3, signal transducers and activators of transcription; TIMP-2, tissue inhibitor of metalloproteinases-2; TSP-1, thrombospondin-1; uPA, urokinase-type plasminogen activator.

## Supplementary Materials

Supplementary Materials
* Supplemental Method*: the complete search algorithm for PubMed and Cochrane.* Supplemental Table I*: quality scores of included studies using Newcastle-Ottawa Scale (maximum score of 9).* Supplemental Table II*: numbers of studies evaluating prognostic value of microRNAs in glioma.* Supplemental Table III*: subgroup analyses of microRNAs and prognosis of glioma.* Supplemental Figure I*: flow diagram of the search process.
