# Supplementary material for: Prognostic Significance of MicroRNAs in Glioma: A Systematic Review and Meta-Analysis
Source: Biomed Res Int. 2019 Mar 26;2019:4015969. doi: 10.1155/2019/4015969 (PMC6457304; doi:10.1155/2019/4015969)

**ONLINE SUPPLEMENT**

**Title:** Prognostic significance of MicroRNAs in glioma: a systematic review and meta-analysis

**Author list:** Yanming Zhang, MB; Jigang Chen, MD; Qiang Xue, MD; Junyu Wang, MD; Liang Zhao, MD; Kaiwei Han, MD; Danfeng Zhang, MD; Lijun Hou, MD.

**Supplementary Method**

The complete search algorithms for PubMed and Cochrane library are as follows.

**PubMed:**

Search (((((((((microrna[MeSH Terms]) OR micrornas[MeSH Terms]) OR micro rna) OR mirna[MeSH Terms]) OR mirnas[MeSH Terms]) OR mir) OR rna, micro[MeSH Terms]) OR micro rnas)) AND ((((((gliomas[MeSH Terms]) OR glioma[MeSH Terms]) OR glial cell tumor[MeSH Terms]) OR glial cell tumors[MeSH Terms]) OR glioblastoma[MeSH Terms]) OR glioblastomas[MeSH Terms])1160

**Cochrane:**

#1"microRNA" or microRNAs or "micro RNA" or micro RNAs or "miRNA" (Word variations have been searched)612

#2 #1 or miRNAs or miR964

#3"glioma" or gliomas or glial cell tumor or glial cell tumors or "glioblastoma" (Word variations have been searched)1856

#4 #3 or glioblastomas1860

#5 #2 and #4

| **Supplementary Table I.** Quality scores of included studies using Newcastle-Ottawa Scale (maximum score of 9) | | | | | | | | | |
| --- | --- | --- | --- | --- | --- | --- | --- | --- | --- |
| case-control studies | | | | | | | | | |
|  | Selection | | | | Comparability | outcome | | | |
| Reference | Adequate definition of cases | Representativeness of cases | Selection of controls | Definition of controls | Comparability on the basis of the design or analysis | Ascertainment of exposure | Same method of ascertainment for cases and controls | Non-response rate (<20%) | Overall |
| Jiang, L 2010 | 1 | 1 | 0 | 1 | 2 | 1 | 1 | 1 | 8 |
| Guan, Y 2010 | 1 | 1 | 0 | 1 | 2 | 1 | 1 | 1 | 8 |
| Zhi, F 2010 | 1 | 0 | 0 | 1 | 1 | 1 | 1 | 1 | 6 |
| Srinivasan, S 2011 | 1 | 0 | 1 | 1 | 1 | 1 | 1 | 1 | 7 |
| Lakomy, R 2011 | 1 | 1 | 0 | 1 | 2 | 1 | 1 | 1 | 8 |
| Lu, S 2012 | 1 | 1 | 1 | 1 | 2 | 1 | 1 | 1 | 9 |
| Hermansen, S 2012 | 1 | 1 | 0 | 1 | 2 | 1 | 1 | 1 | 8 |
| Qiu, S 2013 | 1 | 0 | 1 | 1 | 1 | 1 | 1 | 1 | 7 |
| Zhao, S 2013 | 1 | 1 | 0 | 1 | 2 | 1 | 1 | 1 | 8 |
| Wu, L 2013 | 1 | 1 | 0 | 1 | 2 | 1 | 1 | 1 | 8 |
| Barbano, R 2014 | 1 | 1 | 1 | 1 | 2 | 1 | 1 | 1 | 9 |
| Kim, J 2014 | 1 | 0 | 0 | 1 | 1 | 1 | 1 | 1 | 6 |
| Sun, J 2014 | 1 | 1 | 0 | 1 | 2 | 1 | 1 | 1 | 8 |
| Liu, Q 2014 | 1 | 1 | 1 | 1 | 2 | 1 | 1 | 1 | 9 |
| Men, D 2014 | 1 | 1 | 1 | 1 | 1 | 1 | 1 | 1 | 8 |
| Lai, N 2014 | 1 | 1 | 0 | 1 | 2 | 1 | 1 | 1 | 8 |
| Chen, T 2015 | 1 | 1 | 0 | 1 | 2 | 1 | 1 | 1 | 8 |
| Lai, N 2015 | 1 | 1 | 1 | 1 | 2 | 1 | 1 | 1 | 9 |
| Ji, Y 2015 | 1 | 1 | 0 | 1 | 2 | 1 | 1 | 1 | 8 |
| Pang, C 2015 | 1 | 1 | 0 | 1 | 2 | 1 | 1 | 1 | 8 |
| Sun, G 2015 | 1 | 1 | 0 | 1 | 2 | 1 | 1 | 1 | 8 |
| Guan, Y 2015 | 1 | 1 | 0 | 1 | 2 | 1 | 1 | 1 | 8 |
| Chen, W 2016 | 1 | 1 | 0 | 1 | 2 | 1 | 1 | 1 | 8 |
| Zhang, X 2016 | 1 | 1 | 0 | 1 | 2 | 1 | 1 | 1 | 8 |
| Xiao, Y 2016 | 1 | 1 | 1 | 1 | 2 | 1 | 1 | 1 | 9 |
| Zhang, R 2016 | 1 | 1 | 1 | 1 | 1 | 1 | 1 | 1 | 8 |
| Li, X 2016 | 1 | 1 | 0 | 1 | 2 | 1 | 1 | 1 | 8 |
| Chen, Y 2017 | 1 | 0 | 0 | 1 | 1 | 1 | 1 | 0 | 5 |
| Xue, L 2017 | 1 | 1 | 0 | 1 | 2 | 1 | 1 | 1 | 8 |
| Zhao, H 2017 | 1 | 1 | 1 | 1 | 1 | 1 | 1 | 1 | 8 |
| Sun, C 2017 | 1 | 1 | 0 | 1 | 1 | 1 | 1 | 1 | 7 |

0=”No”, “Unable to determine” or “Not available”.

| **Supplementary Table II:** Numbers of studies evaluating prognostic value of microRNAs in glioma | | | | | | | |
| --- | --- | --- | --- | --- | --- | --- | --- |
| microRNA | N | microRNA | N | microRNA | N | microRNA | N |
| 7 | 1 | 127 | 1 | 203 | 1 | 377 | 1 |
| 9 | 1 | 128a | 1 | 205 | 2 | 378 | 1 |
| 10b* | 3 | 128b | 1 | 210* | 5 | 379 | 1 |
| 15a | 2 | 129 | 1 | 214 | 1 | 383 | 1 |
| 15b* | 5 | 130b | 1 | 215 | 1 | 410 | 1 |
| 16 | 1 | 132 | 1 | 218 | 2 | 429 | 1 |
| 17* | 5 | 134 | 1 | 219 | 1 | 432 | 1 |
| 20a* | 4 | 136 | 1 | 221* | 7 | 454 | 1 |
| 21* | 6 | 137 | 1 | 222* | 7 | 487b | 1 |
| 22 | 1 | 138 | 2 | 223 | 1 | 491 | 1 |
| 23 | 1 | 139 | 1 | 224 | 2 | 497 | 1 |
| 25 | 1 | 142 | 1 | 299 | 1 | 504 | 1 |
| 27a | 1 | 145 | 1 | 320 | 1 | 548a | 1 |
| 27 | 1 | 146b | 2 | 323 | 2 | 584 | 1 |
| 29 | 1 | 148a* | 3 | 326 | 1 | 629-3p | 1 |
| 31 | 2 | 149 | 2 | 328 | 2 | 630 | 1 |
| 34 | 1 | 154 | 1 | 329 | 1 | 637 | 1 |
| 92 | 1 | 155* | 4 | 330 | 1 | 650 | 1 |
| 93 | 1 | 181 | 2 | 335 | 1 | 663 | 1 |
| 105 | 1 | 182* | 4 | 338 | 1 | 675 | 1 |
| 106a* | 6 | 185 | 2 | 340 | 2 | 769 | 1 |
| 106b | 1 | 193a | 1 | 342 | 1 | 922 | 1 |
| 107 | 1 | 195 | 1 | 367 | 1 | 1296 | 1 |
| 124* | 3 | 196* | 4 | 371a | 1 |  |  |
| 125b | 2 | 199a-3p | 1 | 372 | 1 |  |  |
| 126 | 1 | 200b* | 3 | 375 | 1 |  |  |
| *Numbers equal or larger than 3 | | | | | | | |

| **Supplementary Table III:** Subgroup analyses of microRNAs and prognosis of glioma. | | | | | | | | | | |
| --- | --- | --- | --- | --- | --- | --- | --- | --- | --- | --- |
| miR-10b and prognosis | | | | | | miR-15b and prognosis | | | | |
| Subgroups | No. of studies | HR (95% CI) | *P* for test | *I^2^*(%) | P for heterogeneity | No. of studies | HR (95% CI) | *P* for test | *I^2^*(%) | *P* for heterogeneity |
| Type of outcomes |  |  |  |  |  |  |  |  |  |  |
| OS | 2 | 3.70 [2.40, 5.70] | <0.05 | 0 | 0.533 | 4 | 2.06 [0.92, 4.62] | >0.05 | 82 | 0.001 |
| DFS | 1 | 0.93 [0.87, 1.85] | >0.05 | - | - | 1 | 1.32 [1.17, 1.50] | <0.05 | - | - |
| Data sources |  |  |  |  |  |  |  |  |  |  |
| I | 2 | 1.01 [0.81, 1.25] | >0.05 | 77.6 | 0.004 | 2 | 1.30 [1.13, 1.49] | <0.05 | 17 | 0.306 |
| II | - | - | - | - | - | 1 | 1.87 [0.68, 5.15] | >0.05 | - | - |
| III | 1 | 3.42 [2.08, 5.62] | <0.05 | - | - | 2 | 3.42 [1.36, 8.60] | <0.05 | 78.6 | 0.031 |
| miR-17 and prognosis | | | | | | miR-20a and prognosis | | | | |
| Subgroups | No. of studies | HR (95% CI) | *P* for test | *I^2^*(%) | P for heterogeneity | No. of studies | HR (95% CI) | *P* for test | *I^2^*(%) | *P* for heterogeneity |
| Type of outcomes |  |  |  |  |  |  |  |  |  |  |
| OS | 4 | 0.99[0.63,1.55] | >0.05 | 81.5 | 0.001 | 3 | 0.81[0.56,1.16] | >0.05 | 73.1 | 0.024 |
| DFS | 1 | 0.97[0.86,1.09] | >0.05 | - | - | 1 | 1.03[0.93,1.14] | >0.05 | - | - |
| Data sources |  |  |  |  |  |  |  |  |  |  |
| I | 2 | 0.99[0.86,1.16] | >0.05 | 36.8 | 0.191 | 2 | 1.05[0.94,1.17] | >0.05 | 9.4 | 0.346 |
| II | 2 | 0.67[0.56,0.79] | <0.05 | 0 | 0.8 | 2 | 0.68[0.57,0.80] | <0.05 | 0 | 0.933 |
| III | 1 | 2.14[1.06,4.31] | <0.05 | - | - | - | - | - | - | - |
| miR-21 and prognosis | | | | | | miR-106a and prognosis | | | | |
| Subgroups | No. of studies | HR (95% CI) | *P* for test | *I^2^*(%) | P for heterogeneity | No. of studies | HR (95% CI) | *P* for test | *I^2^*(%) | *P* for heterogeneity |
| Type of outcomes |  |  |  |  |  |  |  |  |  |  |
| OS | 5 | 1.59[0.97,2.61] | >0.05 | 88.7 | 0.001 | 5 | 0.66[0.50,0.87] | <0.05 | 59.5 | 0.030 |
| DFS | 1 | 1.44[1.26,1.63] | <0.05 | - | - | 1 | 1.04[0.94,1.16] | >0.05 | - | - |
| Data sources |  |  |  |  |  |  |  |  |  |  |
| I | 5 | 1.66[1.33,2.06] | <0.05 | 83.6 | 0.001 | 4 | 0.89[0.71,1.11] | >0.05 | 71.0 | 0.002 |
| II | 1 | 0.57[0.21,1.53] | >0.05 | - | - | 2 | 0.65[0.54,0.78] | <0.05 | 0 | 0.809 |
| III | - | - | - | - | - | - | - | - | - | - |
| miR-124 and prognosis | | | | | | miR-148a and prognosis | | | | |
| Subgroups | No. of studies | HR (95% CI) | *P* for test | *I^2^*(%) | P for heterogeneity | No. of studies | HR (95% CI) | *P* for test | *I^2^*(%) | *P* for heterogeneity |
| Type of outcomes |  |  |  |  |  |  |  |  |  |  |
| OS | 2 | 1.25[0.35,4.43] | >0.05 | 86.1 | 0.007 | 2 | 1.20[1.12,1.28] | <0.05 | 0 | 0.816 |
| DFS | 1 | 0.81[0.77,0.86] | <0.05 | - | - | 1 | 1.04[0.91,1.20] | >0.05 | - | - |
| Data sources |  |  |  |  |  |  |  |  |  |  |
| I | 2 | 0.81[0.77,0.86] | <0.05 | 0 | 0.663 | 2 | 1.09[0.97,1.23] | >0.05 | 65.5 | 0.033 |
| II | 1 | 2.37[1.24,4.53] | <0.05 | - | - | 1 | 1.21[1.08,1.36] | <0.05 | - | - |
| III | - | - | - | - | - | - | - | - | - | - |
| miR-155 and prognosis | | | | | | miR-182 and prognosis | | | | |
| Subgroups | No. of studies | HR (95% CI) | *P* for test | *I^2^*(%) | P for heterogeneity | No. of studies | HR (95% CI) | *P* for test | *I^2^*(%) | *P* for heterogeneity |
| Type of outcomes |  |  |  |  |  |  |  |  |  |  |
| OS | 3 | 1.20 [0.80,1.86] | >0.05 | 89.9 | 0.001 | 4 | 1.21 [0.71,2.05] | >0.05 | 81.0 | 0.001 |
| DFS | 1 | 1.10 [0.97,1.25] | >0.05 | - | - | - | - | - | - | - |
| Data sources |  |  |  |  |  |  |  |  |  |  |
| I | 3 | 1.06 [0.90,1.24] | >0.05 | 64.3 | 0.024 | 3 | 0.95 [0.72,1.26] | >0.05 | 16.8 | 0.307 |
| II | - | - | - | - | - | - | - | - | - | - |
| III | 1 | 1.14 [0.94,1.39] | >0.05 | - | - | 1 | 3.39 [1.98,5.80] | <0.05 | - | - |
| miR-196 and prognosis | | | | | | miR-200b and prognosis | | | | |
| Subgroups | No. of studies | HR (95% CI) | *P* for test | *I^2^*(%) | P for heterogeneity | No. of studies | HR (95% CI) | *P* for test | *I^2^*(%) | *P* for heterogeneity |
| Type of outcomes |  |  |  |  |  |  |  |  |  |  |
| OS | 4 | 1.88 [1.03,3.41] | <0.05 | 77.5 | 0.001 | 3 | 1.11 [0.45,2.74] | >0.05 | 77.5 | 0.012 |
| DFS | - | - | - | - | - | - | - | - | - | - |
| Data sources |  |  |  |  |  |  |  |  |  |  |
| I | 2 | 1.37 [0.61,3.05] | >0.05 | 82.6 | 0.003 | - | - | - | - | - |
| II | 1 | 3.37 [1.20,9.46] | <0.05 | - | - | 2 | 1.66 [0.73,3.78] | >0.05 | 71.3 | 0.062 |
| III | 1 | 3.17 [1.82,5.53] | <0.05 | - | - | 1 | 0.30 [0.09,0.98] | <0.05 | - | - |
| miR-210 and prognosis | | | | | | miR-221 and prognosis | | | | |
| Subgroups | No. of studies | HR (95% CI) | *P* for test | *I^2^*(%) | P for heterogeneity | No. of studies | HR (95% CI) | *P* for test | *I^2^*(%) | *P* for heterogeneity |
| Type of outcomes |  |  |  |  |  |  |  |  |  |  |
| OS | 4 | 1.55[0.93,2.57] | >0.05 | 91.8 | 0.001 | 6 | 1.48[1.01,2.15] | <0.05 | 84.9 | 0.001 |
| DFS | 1 | 1.06[0.98,1.16] | >0.05 | - | - | 1 | 1.14[1.02,1.26] | <0.05 | - | - |
| Data sources |  |  |  |  |  |  |  |  |  |  |
| I | 4 | 1.14[0.93,1.39] | >0.05 | 82.3 | 0.001 | 5 | 1.39[1.14,1.68] | <0.05 | 62.7 | 0.013 |
| II | - | - | - | - | - | 2 | 0.95[0.52,1.72] | >0.05 | 94.0 | 0.001 |
| III | 1 | 2.30[1.47,3.60] | <0.05 | - | - | - | - | - | - | - |
| miR-222 and prognosis | | | | | |  |  |  |  |  |
| Subgroups | No. of studies | HR (95% CI) | *P* for test | *I^2^*(%) | P for heterogeneity |  |  |  |  |  |
| Type of outcomes |  |  |  |  |  |  |  |  |  |  |
| OS | 6 | 1.31 [0.86,2.00] | >0.05 | 86.6 | 0.001 |  |  |  |  |  |
| DFS | 1 | 0.99 [0.84,1.16] | >0.05 | - | - |  |  |  |  |  |
| Data sources |  |  |  |  |  |  |  |  |  |  |
| I | 5 | 1.19 [0.95,1.49] | >0.05 | 78.2 | 0.001 |  |  |  |  |  |
| II | 2 | 0.87 [0.42,1.84] | >0.05 | 95.1 | 0.001 |  |  |  |  |  |
| III | - | - | - | - | - |  |  |  |  |  |
| CI: confidence interval; HR: hazard ratio. OS: overall survival; DFS: disease free survival; I: data extract directly; II: data calculated from HR and P value; III: data calculated from survival curve | | | | | |  |  |  |  |  |

**Supplementary Figure I:** Flow diagram of the search process


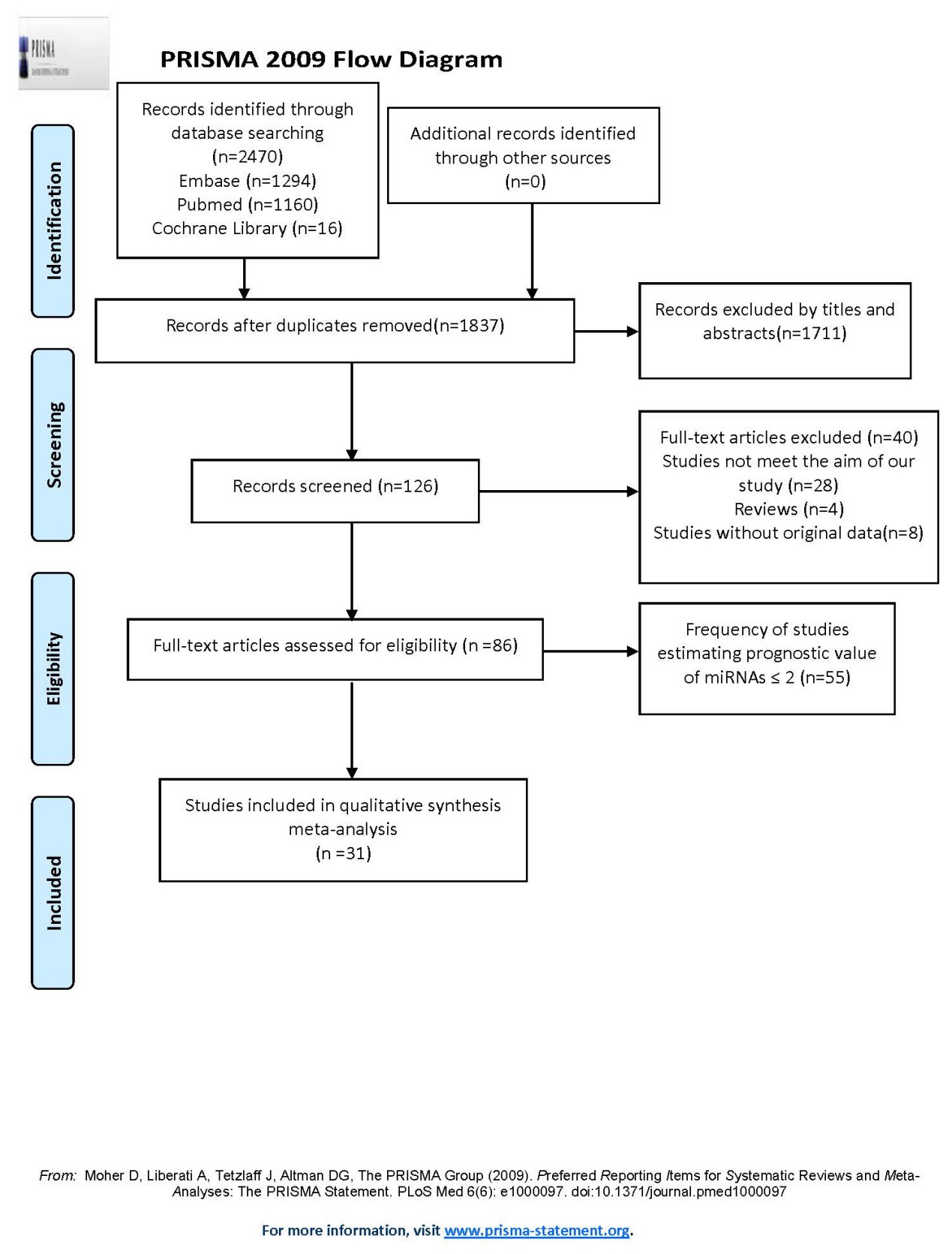

Supplement: Supplementary Materials — Supplemental Method: the complete search algorithm for PubMed and Cochrane. Supplemental Table I: quality scores of included studies using Newcastle-Ottawa Scale (maximum score of 9). Supplemental Table II: numbers of studies evaluating prognostic value of microRNAs in glioma. Supplemental Table III: subgroup analyses of microRNAs and prognosis of glioma. Supplemental Figure I: flow diagram of the search process. [file 4015969.f1.docx]
